# The scale of neurodegeneration in moderate-to-severe traumatic brain injury: a systematic review protocol

**DOI:** 10.1186/s13643-019-1208-0

**Published:** 2019-12-18

**Authors:** Bhanu Sharma, Alana T. Changoor, Leanne Monteiro, Brenda Colella, Robin E. A. Green

**Affiliations:** 1 University Health Network – Toronto Rehabilitation Institute, 550 University Avenue, Toronto, ON M5G2A2 Canada; 2 Department of Medical Sciences, McMaster University, 1280 Main Street West, Hamilton, ON L8S 4 L8 Canada; 3 Department of Psychiatry, University of Toronto, 550 University Avenue, Toronto, ON M5G2A2 Canada

**Keywords:** Traumatic brain injury, TBI, Neurodegeneration, MRI, Neuroimaging, Rehabilitation

## Abstract

**Background:**

Our understanding of recovery after moderate-to-severe traumatic brain injury (TBI) has shifted. Until recently, it was presumed that following a period of acute neurological vulnerability, the brain remained stable in the chronic stages of injury. However, recent research has shown *neurodegeneration* in the chronic stages of moderate-to-severe TBI, challenging the assumption of neurological stability. While there is extensive evidence that neurodegeneration occurs, debate remains regarding the scale and timing. This systematic review will evaluate the scale and timelines of neurodegeneration in adult patients with moderate-to-severe TBI.

**Methods:**

Literature searches will be conducted in six electronic databases (from inception onwards), including MEDLINE, EMBASE, PsycINFO, CINAHL, SportDiscus, and Cochrane Central Register of Controlled Trials. We will include observational studies that examine neurodegenerative changes within a single sample of TBI patients or studies that compare neuroimaging outcomes between TBI patients and healthy controls. Our primary outcome is structural neuroimaging, and our secondary outcome is diffusion tensor imaging for detection of post-injury white matter changes. All screening, data extraction, and study quality appraisal will be performed independently by the same two study members. It is expected that a narrative summary of the literature will be produced. If feasible, we will conduct a random-effects meta-analysis. However, given the expected heterogeneity between studies (with respect to, for example, timing of imaging, regions imaged) we do not expect to perform a meta-analysis; rather, a narrative synthesis of our findings is expected to be performed.

**Discussion:**

Understanding the scale and timelines of neurodegeneration in moderate-to-severe TBI (as well as which brain areas are most vulnerable to chronic declines) can inform intervention research designed to offset such changes. This may help improve patient outcome following moderate-to-severe TBI and, in turn, reduce the burden of the injury.

**Systematic review registration:**

PROSPERO CRD42019117548

## Background

Moderate-to-severe traumatic brain injury (TBI) is a leading public health concern [12, 13]. The clinical presentation of moderate-to-severe TBI is diverse and can include a broad range of cognitive and behavioral impairments that may persist indefinitely [5, 11, 19, 24, 29]. More than 1% of the general population is estimated to be living with disability secondary to TBI [29] and TBI is projected to become the third-most cause of disability by the year 2020 [1]. Further, rates of TBI are rising [19], suggesting that the burden of injury is vast yet still increasing.

Until recently, neurological stability in the sub-acute and chronic stages of moderate-to-severe TBI was presumed. However, recent studies have challenged this notion, and there is now a compelling evidence base that suggests that TBI is neurodegenerative [2, 4, 6, 9, 16, 25–27]. More specifically, progressive atrophy of the brain has been documented in TBI by multiple research groups [2–4, 6–9, 16, 25, 27] along with reduced white matter integrity [3, 4, 7, 22]. Such degenerative changes have been negatively associated with neuropsychological correlates (e.g., executive control, working memory) and with clinical correlates (e.g., anxiety [7, 22, 23, 27]).

While the presence of neurodegeneration in TBI has been established, study findings vary with respect to several key parameters. For example, with regard to scale, our group has reported neurodegenerative changes in more than 95% of moderate-to-severe TBI patients [9], whereas others have reported lower rates. The timelines of neurodegenerative changes have also varied across studies, and an assessment of the brain regions that are particularly vulnerable to post-acute atrophy has yet to be performed. A recent literature review by Harris et al. [10] examined neurodegeneration in TBI and found that atrophy can occur at a rate of 5% per year, although this estimate is based on studies where imaging was performed acutely, and may thus be confounded by resolution of edema. Further research is still needed to establish post-acute timelines of neurodegeneration based on longitudinal data. To date, evidence on the scale and timelines of neurodegeneration in the post-acute stages of TBI has not been consolidated through systematic review.

A systematic review that synthesizes and appraises the literature on prevalence and timelines of neurodegenerative changes in TBI, as well as the most implicated brain regions, would inform future intervention research. More specifically, it would help to establish the need for intervention research designed to offset neurodegeneration in chronic moderate-to-severe TBI, and it would help to determine the timing of intervention as well as which region(s) to target. Therefore, this systematic review will help establish the scale and timelines of whole brain and regional (i) volumetric brain changes and (ii) reductions in white matter integrity in adults with moderate-to-severe TBI patients who are in the post-acute stages of injury.

## Methods/design

### Guidelines and registration

This review protocol was reported in accordance with reporting guidance provided by the Preferred Reporting Items for Systematic Reviews and Meta-Analysis Protocols (PRISMA-P) statement [21] (see PRISMA-P checklist, provided as a Additional file 1). Our systematic review will be reported in accordance with the Preferred Reporting Items for Systematic Reviews and Meta-Analysis (PRISMA) statement [15].

In addition, at the time of performing preliminary searches (i.e., before piloting our formal searches and screening any articles), the review was registered with the International Prospective Register of Systematic Reviews (PROSPERO CRD42019117548). It should be noted that any changes to our protocol will be submitted as amendments to PROSPERO.

### Eligibility criteria

Study eligibility will be assessed using a PICO framework [20], as detailed below.

#### Participants

We will include studies on adults (aged 18–65) with moderate-to-severe TBI in the post-acute stages of injury. Injury severity is typically defined using measures of consciousness such as the Glasgow Coma Scale (where a score < 13 on the 15-point scale constitutes a moderate or severe TBI), length of post-traumatic amnesia (PTA; where duration of PTA greater than one day is considered moderate severity or greater) or length of loss of consciousness (LOC; where duration of LOC > 1 h is considered moderate severity or greater). Studies that exclusively examine patients with milder brain injuries (e.g., concussion) will be excluded. Further, studies in animal models will be excluded.

#### Intervention

Our systematic review is of observational studies (that examine the natural progression of neurodegeneration in TBI), and not randomized controlled trials. It is expected that most studies included in our review will be longitudinal cohort studies or cross-sectional studies comparing neuroimaging outcomes between TBI patients and healthy controls. Further, baseline neuroimaging must take at approximately 2 months post-injury or later; there is no cap on time post-injury on any subsequent neuroimaging assessments as our goal is to characterize neurodegenerative changes that commence anytime within the sub-acute to chronic stages of TBI.

#### Comparator

Studies with either a within-group comparison (i.e., single cohort of moderate-to-severe TBI patients measured longitudinally) or between-group comparison (namely a comparison between TBI patients and healthy controls from the general population without a history of TBI or other relevant neurological impairment) will be included. The use of these two comparison groups will allow our systematic review to assess the natural course of neurodegeneration (within-groups) as well as differences in brain volume/white matter integrity between TBI patients and healthy controls.

#### Outcome

Eligible studies must employ neuroimaging to examine volumetric brain changes (e.g., T1- or T2-weighted images) and/or changes in white matter integrity (as assessed using diffusion tensor imaging). Volumetric brain changes are our primary outcome, as the neurodegenerative effects of TBI are often best observed and documented using imaging that can visualize structural brain changes. Our secondary outcome is diffusion tensor imaging, given that fewer studies have examined neurodegeneration using this outcome and it cannot detect degenerative changes in non-white matter structures (such as the hippocampi), which are highly susceptible to neurodegeneration following TBI. For characterization purposes, we will also collect information on demographic (e.g. age, sex, years of education) and injury (e.g. mechanism of injury, time post-injury, severity of injury) variables.

### Information sources

We will systematically search, from inception onwards, MEDLINE (OVID), EMBASE (OVID), PsycINFO (OVID), CINAHL (EBSCO), SportDiscus (EBSCO), and Cochrane Central Register of Controlled Trials. The reference lists of examined full-text papers will be scrutinized for additional relevant articles. Further, as per a Google Scholar search, studies that cited the articles included in our review will also be evaluated for inclusion (based on a title screen) by two reviewers. Relevant articles identified by these means will be then be reviewed in full for inclusion. Hardcopies of articles not available online will be requested from the university library, but otherwise electronically retrieved.

### Search strategy

The first draft of each search will be developed by team members with experience in brain injury and systematic reviews, using medical subject headings (MeSH) and text keywords. The searches will be reviewed and revised by a health sciences librarian, and optimized for each health sciences database. Limitations will not be placed on the year of publication. Only English language articles will be retrieved.

Weekly automated email updates will provide our team with a list of recently published articles that were identified by our searches. These updates will be forwarded to the two reviewers, who will screen titles of the articles retrieved through these weekly updates to determine whether any should undergo full-text screening. The purpose of these automated updates is to ensure our review is current and updated weekly (as necessary).

A sample search strategy has been included as a Additional file 2:

### Data management

Search results from each database will be exported from each search interface as .ris files. The files will then be imported into EndNote x9 and collated. Duplicates will then be removed in EndNote x9, and the collated, de-duped list of articles will be imported into "abstrackr" [28], an online tool that facilitates study screening. More specifically, abstrackr enables a reviewer to screen articles while remaining blind to the selections of their reviewing partner, and it automatically identifies articles over which there is discrepancy regarding inclusion or exclusion.

### Screening and selection process

Two members of the study team (AC and LM) will screen all articles identified from the search independently.

First, in abstrackr, titles and abstracts returned from initial searches will be screened based on the eligibility criteria outlined above. Second, full-text articles will be examined in detail and screened for eligibility. Third, references of all considered articles will be hand-searched to identify any relevant report missed in the search strategy by two reviewers independently. Any disagreement between reviewers will be resolved by discussion to meet a consensus.

### Data extraction

Data extraction forms will be developed by the study team and piloted prior to full-text data extraction. The two reviewers who completed article screening will then each independently populate a data extraction form, with data from the final pool of eligible articles. To ensure consistency in data extraction, the two independently completed forms will then be reviewed by another team member (BS), who will check for discrepancies between the two forms as well as for discrepancies between the completed forms and the original full-text articles. If discrepancies are present, they will be resolved through re-review of the article(s) in question. If there are any uncertainties with respect to the data presented in eligible articles, the original study authors will be contacted by email and clarification regarding the data or how it is presented will be requested.

Data extraction forms will contain fields specific to study and population characteristics as well as outcome measures. The full list of these characteristics is available in a Additional file 3.

### Assessment of study quality

Given that our review is one of observational research, we will use the NIH’s Quality Assessment Tool for Observational Cohort and Cross-Sectional Studies [17]. At the study level, each reviewer will independently evaluate each article using this tool. Another team member (BS) will review quality assessment ratings generated by each reviewer on an article-to-article basis to ensure consistency. Any discrepancies in quality evaluation will be resolved through discussion by the two reviewers, and re-evaluation of the original article as necessary. When performing our data synthesis, we will comment on data quality; the NIH Quality Assessment Tool for Observational and Cohort and Cross-Sectional Studies permits categorization of study quality as Good, Fair, or Poor, per their guidelines. Therefore, this tool will help us comment on the strength of our evidence base (and thus findings) and in what areas additional research may be needed.

### Data synthesis

Given the high probability that our data will not be sufficiently homogeneous for meta-analysis (given that patients from different studies may be imaged at different times post-injury, and neuroimaging may be of different brain regions), a systematic narrative synthesis is expected, as informed by recent guidelines [18]. More specifically, we will stratify our data so that our narrative synthesis will speak to the effects of TBI on neurodegeneration with respect to (i) whole brain changes, and (ii) regional brain changes, as possible. Within each of these two broad categories, we will speak to the effects of TBI on neurodegeneration as measured using volumetric neuroimaging and diffusion tensor imaging. The timelines of these changes will also be discussed. Study quality ratings will be presented and used to contextualize our findings. If there are sufficient data (i.e., at least 3 studies per sub-group analysis) and data are homogeneous—with respect to, for example, population (injury severity, age, sex), time post-injury (timing of each neuroimaging assessment), type of comparison (within or between groups), and unit of analysis (whole brain, specific sub-regions)—a random-effects meta-analysis will be performed. Heterogeneity will be assessed with chi-square tests as well as *I*^2^ tests. If heterogeneity is deemed excessive (*p* < 0.1 on chi-square or *I*^2^ > 50%), data will be analyzed narratively.

If a meta-analysis is performed, for continuous measures (i.e., neuroimaging of brain volume or white matter integrity), weighted means (with 95% CI) will be used to understand the magnitude of neurodegeneration. Sub-group analyses will be performed (if data can be pooled) to assess neurodegenerative changes in different brain regions.

## Discussion

Our understanding of TBI has changed. This injury is no longer considered an acute event with a circumscribed period of recovery. Rather, accumulating findings indicate that TBI is more akin to a neurodegenerative disease, with chronic post-injury neurodegeneration now widely documented in many studies [9]. However, the scale and timelines of this neurodegeneration have not been established through systematic review.

Our systematic review will be performed to address this critical knowledge gap. In doing so, our review will be begin to quantify the proportion of moderate-to-severe TBI patients that experience neurodegeneration, and, therefore, how many may require additional intervention. Understanding the scope of the problem can inform resources needed to manage the progression of symptoms, and the need to develop, test, and implement interventions for these chronic sequelae of TBI. Further, knowing which brain regions experience the most degeneration can assist in developing more targeted interventions, for example, those designed to mitigate chronic neurodegeneration of memory centers (i.e., hippocampi) through environmental enrichment (which is negatively associated with the magnitude of neurodegeneration [14]). An anticipated limitation of this review is heterogeneity between study samples (with respect to, for example, time post-injury at imaging assessment and brain regions studied), which may preclude meta-analysis. Nonetheless, establishing the scale and timelines of neurodegeneration in moderate-to-severe TBI is a necessary first step towards improving treatment and management of chronic brain injury.

## Supplementary information

**Additional file 1.** PRISMA-P 2015 Checklist.

**Additional file 2.** Sample search Strategy for Medline (OVID interface, 1946-Present).

**Additional file 3:** **Table S1.** Primary and secondary outcomes collected on our extraction form.

## Data Availability

The datasets used and/or analyzed during the current study are available from the corresponding author on reasonable request.

## Abbreviations

GCS: Glasgow Coma Scale
PRISMA: Preferred Reporting Items for Systematic Reviews and Meta-Analysis Protocols
PROSPERO: International Prospective Register of Systematic Reviews
PTA: Post-traumatic amnesia
TBI: Traumatic brain injury
