# Supplementary material for: The scale of neurodegeneration in moderate-to-severe traumatic brain injury: a systematic review protocol
Source: Syst Rev. 2019 Dec 18;8:332. doi: 10.1186/s13643-019-1208-0 (PMC6921548; doi:10.1186/s13643-019-1208-0)
Supplement: Supplementary file 2 — Additional file 2. Sample search Strategy for Medline (OVID interface, 1946-Present). [file 13643_2019_1208_MOESM2_ESM.docx]

***Sample search Strategy for Medline (OVID interface, 1946-Present)***

*1. (volum* loss adj2 (brain* or cereb* or white matter* or grey matter* or gray matter*)).ti,ab,kw.*

*2. (neuro* adj1 (degener* or deterior* or loss*)).ti,ab,kw.*

*3. (ventricul* adj1 enlarge*).ti,ab,kw.*

*4. (atrophy adj2 (brain* or cerebr* or neuro*)).ti,ab,kw.*

*5. (axon* adj1 (degen* or neurodegen* or damag*)).ti,ab,kw.*

*6. neurodegenerat*.ti,ab,kw.*

*7. demyelin*.ti,ab,kw.*

*8. exp Neurodegenerative Diseases/*

*9. 1 or 2 or 3 or 4 or 5 or 6 or 7 or 8*

*10. (head adj1 (injur* or trauma*)).ti,ab,kw.*

*11. (brain adj1 (injur* or trauma* or damag*)).ti,ab,kw.*

*12. TBI*.ab,ti,kw.*

*13. exp Brain Injuries/*

*14. 10 or 11 or 12 or 13*

*15. 9 and 14*

*16. exp animal/ not human.sh.*

*17. 15 not 16*

*18. limit 17 to English language*
