# Supplementary material for: The scale of neurodegeneration in moderate-to-severe traumatic brain injury: a systematic review protocol
Source: Syst Rev. 2019 Dec 18;8:332. doi: 10.1186/s13643-019-1208-0 (PMC6921548; doi:10.1186/s13643-019-1208-0)
Supplement: Supplementary file 3 — Additional file 3: Table S1. Primary and secondary outcomes collected on our extraction form. [file 13643_2019_1208_MOESM3_ESM.docx]

**TABLES**

| Extraction category | Fields |
| --- | --- |
| Study characteristics | Author |
|  | Title |
|  | Journal |
|  | Year of Publication |
|  | Country of Publication |
|  | Design |
|  | Sample size [total & subgroups] |
|  | Control variables [if applicable] |
|  | Intervention [frequency, intensity, time, type] |
|  | Study quality [Grade] |
| Population characteristics | Mean age of sample [total & subgroups] |
|  | Time post-injury [total & subgroups] |
|  | Sex (% male) [total & subgroups] |
|  | Injury severity (GCS, PTA) [total & subgroups] |
|  | Mechanism of injury [total & subgroups] |
|  | Baseline characterization |
|  | Time elapsed between injury and baseline [total & subgroups] |
|  | Time elapsed between baseline and follow-up [total & subgroups] |
| Outcomes - Primary | Baseline imaging [imaging type, regions assessed, sequence parameters] – both whole brain and regional volumetric or white matter changes will be assessed |
|  | Follow-up imaging [imaging type, regions assessed, sequence parameters] – both whole brain and regional changes will be assessed |
| Outcomes – Secondary | Baseline predictors of neurodegeneration |
|  | Follow-up predictors of neurodegeneration |

**Table S1:** Primary and secondary outcomes collected on our extraction form.
